# Challenges and solutions for application and wider adoption of wearable robots

**DOI:** 10.1017/wtc.2021.13

**Published:** 2021-11-21

**Authors:** Jan Babič, Matteo Laffranchi, Federico Tessari, Tom Verstraten, Domen Novak, Nejc Šarabon, Barkan Ugurlu, Luka Peternel, Diego Torricelli, Jan F. Veneman

**Affiliations:** 1 Laboratory for Neuromechanics and Biorobotics, Department of Automation, Biocybernetics and Robotics, Jožef Stefan Institute, Ljubljana, Slovenia; 2 Rehab Technologies Lab, Istituto Italiano di Tecnologia, Genoa, Italy; 3 Robotics & Multibody Mechanics Research Group, Vrije Universiteit Brussel and Flanders Make, Brussels, Belgium; 4 University of Wyoming, Laramie, Wyoming, USA; 5 Faculty of Health Sciences, University of Primorska, Izola, Slovenia; 6 Biomechatronics Laboratory, Faculty of Engineering, Ozyegin University, Istanbul, Turkey; 7 Delft Haptics Lab, Department of Cognitive Robotics, Delft University of Technology, Delft, The Netherlands; 8 Cajal Institute, Spanish National Research Council, Madrid, Spain; 9 Hocoma AG, Volketswil, Switzerland

**Keywords:** exoskeleton, user acceptance, wearable, wearable robotics

## Abstract

The science and technology of wearable robots are steadily advancing, and the use of such robots in our everyday life appears to be within reach. Nevertheless, widespread adoption of wearable robots should not be taken for granted, especially since many recent attempts to bring them to real-life applications resulted in mixed outcomes. The aim of this article is to address the current challenges that are limiting the application and wider adoption of wearable robots that are typically worn over the human body. We categorized the challenges into mechanical layout, actuation, sensing, body interface, control, human–robot interfacing and coadaptation, and benchmarking. For each category, we discuss specific challenges and the rationale for why solving them is important, followed by an overview of relevant recent works. We conclude with an opinion that summarizes possible solutions that could contribute to the wider adoption of wearable robots.

## Introduction

Wearable robots are slowly but persistently entering our everyday lives—either at work, in sports, during rehabilitation, and in other situations. Even if we do not yet use such a robot, we can certainly imagine how nice it would be to wear a device that could reduce the burden of manual work on our body, increase our abilities during sports, or allow us to perform effective rehabilitation at our homes. With continuing advancements in mechanical design, modern materials, improved power sources, miniaturization of electronics, increased rate of data processing, and better understanding of biomechanics, widespread adoption of wearable robots seems to be within reach. Nevertheless, despite all these scientific and technological advancements, many recent pioneering efforts to bring wearable robots to real-life applications were accompanied by mixed feelings of those who were supposed to benefit from them (Hensel and Keil, 2019; Davis et al., 2020; Mortenson et al., 2020).

The aim of this review article is to address the current challenges that are limiting application and wider adoption of wearable robots in various domains (e.g., industry, rehabilitation, sports, healthcare, for physically challenged individuals, harsh environments, and aeronautics) and offer an outlook on relevant solutions and approaches that could overcome these challenges. We focus on wearable robots that are typically worn over the human body—for example, stiff exoskeletons, soft actuated suits, and hybrid soft-stiff exoskeletons. However, we also mention robotic prosthetic devices wherever relevant.

**Figure 1.**
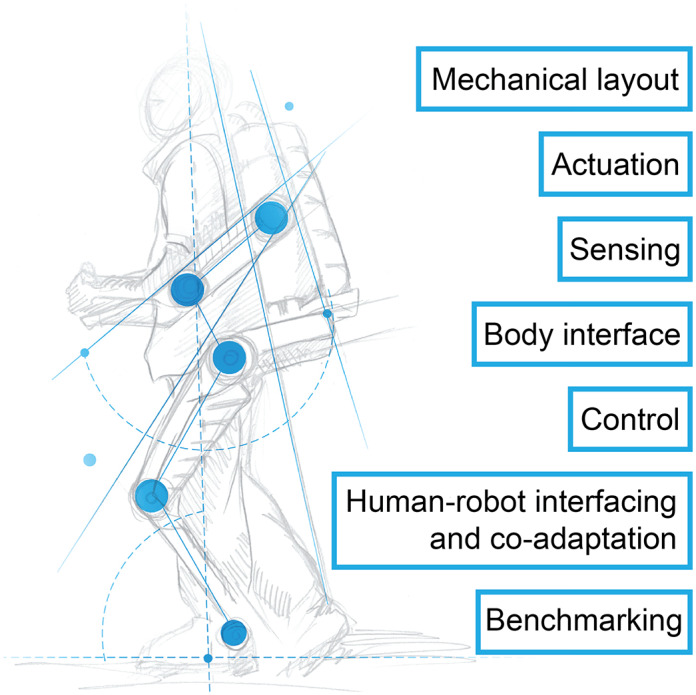
Seven aspects of wearable robots that are addressed in the following sections.

In the following seven sections, we review various aspects of wearable robots and their interaction with the human body (Figure 1). The sections start with the challenges and the rationale for why solving them is important, followed by an overview of relevant recent works related to the specific challenges. Each section then concludes with an opinion that summarizes possible solutions to advance wearable robots beyond the state of art and contribute to their wider adoption.

## Mechanical Layout

Today’s wearable robotic devices feature a diverse variety of mechanical designs depending on the specific task they are intended to perform. Although examples of hybrid designs exist, most design approaches for wearable robots can be classified into two main categories: stiff devices, which consist of rigid, mostly metallic, structural elements, and so-called “exosuits,” which consist of flexible or even textile elements (Sanchez-Villamañan et al., 2019). The main challenge that engineers face in the development of wearable robot mechanics is to realize compact and light structures, combined with suitable kinematics so that the robot does not hinder the human’s musculoskeletal kinematics. In other words, good exoskeleton mechanical design must be able to effectively replicate or support the complex movements of the human limbs while being as unobtrusive as possible in their utilization context and in their management.

### Stiff Exoskeleton Designs

**Figure 2.**
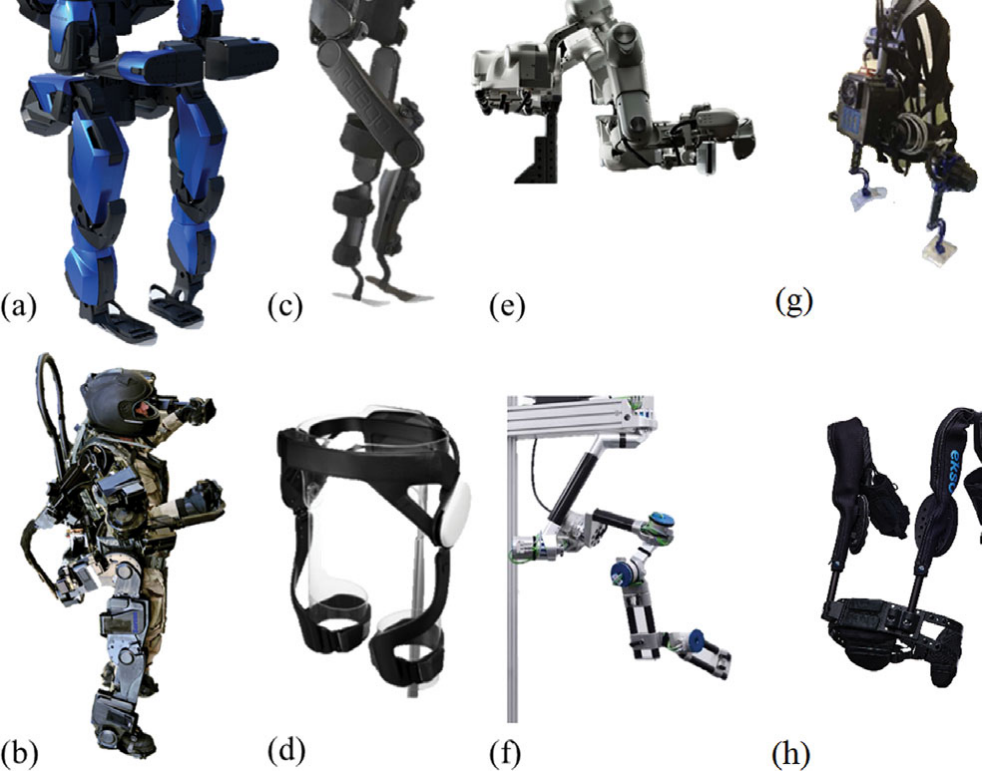
Stiff exoskeletons: (a) the power augmentation exoskeleton Guardian XO from Sarcos (www.sarcos.com), (b) the Raytheon XOS 2 (www.raytheon.com), (c) the lower limb rehabilitation exoskeleton Indego (www.indego.com; Farris et al., 2011; Jyräkoski et al., 2020), (d) assistive lower limb exoskeleton GEMS (Lim et al., 2019), (e) the rehabilitation upper limb Harmony Exoskeleton by ReNeu Lab (www.reneu.robotics.utexas.edu/harmony-exoskeleton; Kim and Deshpande, 2017), (f) rehabilitation upper limb exoskeleton ANYexo from ETH (www.sms.hest.ethz.ch/research/current-research-projects/armin-robot/ANYexo.html; Zimmermann et al., 2019), (g) the occupation active trunk exoskeleton Robo.Mate (Huysamen et al., 2018), and (h) the occupational passive upper limb exoskeleton EksoVest (www.eksobionics.com/ekso-evo/; Kim et al., 2018a,b).

Exoskeletons with stiff structure and active joint actuation are the most widely known wearable devices and will be addressed in this section by analyzing applications for both the upper and lower limb. Depending on the amount of net active torque they can generate, they can be classified as “high-torque” or “low-torque” solutions. Specifically, “high-torque” stiff exoskeletons can generate sufficient torque to realize absolute control over the limb to which the exoskeleton is attached. This subcategory is ideal either when the wearer needs substantial support by the machine or when high levels of controllability are needed on the limb. High-torque use cases include lower limb assistance for severely impaired people, (Esquenazi et al., 2012; Wang et al., 2015; Milia et al., 2016; Vouga et al., 2017; van der Kooij et al., 2019; Jyräkoski et al., 2020; Figure 2c; www.indego.com) or power augmentation applications in which also upper limbs might be involved (Kazerooni, 2005; Marcheschi et al., 2011; www.sarcos.com; www.raytheon.com; Figure 2a,b). While kinematics that enables high levels of controllability of the limb is frequently used in rehabilitation applications since it allows the movement pattern to be precisely tuned to the patient’s needs (Bai and Christensen, 2017; Molteni et al., 2018), power augmentation applications typically do not demand total control of the biological joints. Indeed, within the “high-torque” class of wearable robots, some devices do not constrain the full limb of the wearer and deliberately leave freedom of movement to some biological joints. A similar approach can be found in underconstrained exoskeletons, where dedicated kinematic solutions and redundancy are exploited to maximize the wearability and usability of the device (Lo and Xie, 2012; Tiseni et al., 2019). In fact, nonfully constrained architectures may also be employed in other applications where absolute control of limb joints is not needed, such as assistive/industrial scenarios (Guizzo and Goldstein, 2005; Bogue, 2018). In these applications, biological joints might not exactly match those of the robot, resolving possible joint alignment issues.

In all these high-torque lower/full-body exoskeletons, the mechanical structure needs to be in direct physical contact with the ground (i.e., through the feet) to support the load of the machine and the user.^1^ Although significant engineering efforts have been made to lighten the structures of these devices (Farris et al., 2011; Vouga et al., 2017), their mechanical structure still tends to be cumbersome due to the considerable load they need to bear as well as the high torques that have to be generated and transferred through the structure to the ground, the load, and possibly to human limbs.

On the other side of the spectrum, “low-torque” stiff exoskeletons are not capable of generating sufficient torque to realize total control over either the upper or lower limb, and thus provide only partial assistance to the user. Application domains of this class include gait assistance devices (Lim et al., 2019; Zhang et al., 2019; Figure 2d) and occupational exoskeletons, either active (Figure 2g) or passive (Figure 2h; Bogue, 2018; Huysamen et al., 2018; Theurel and Desbrosses, 2019; www.eksobionics.com/ekso-evo/). The main function of this “low-torque” category is to assist the wearer in performing a specific motor task. As a result of the smaller force/torque exchange required by these applications, their structure is typically slimmer. These “low-torque” exoskeletons are considerably lighter than those in the “high torque” group, and to a certain extent their weight can thus be borne by the user.

Concerning the upper limb, most actuated exoskeletons for this limb are used for robot-assisted rehabilitation (Nef et al., 2007; Gopura et al., 2016; Kim and Deshpande, 2017; Rehmat et al., 2018). In upper-body exoskeletons, the device can be fixed on an external platform (Caldwell et al., 2007; Noda et al., 2014), which severely limits its mobility; conversely, it can be carried entirely by the human (Balasubramanian et al., 2008), which requires sufficient fitness to support the weight.

Upper limb rehabilitation devices need to deliver torque to the wearer during physical exercise (Lo and Xie, 2012; Mehrholz et al., 2012; Wendong et al., 2020), and typically involve designs that allow high levels of controllability of the limb (Nef et al., 2007; Gopura et al., 2016; Bai and Christensen, 2017). The robot is usually used in one of three modes: (a) passive mobilization, where the user relaxes their arm and the robot moves it to improve its range of motion (Brahmi et al., 2019); (b) assist as needed, where the robot assists voluntary movements of the patient (Reinkensmeyer et al., 2012); or (c) resistive or error augmentation mode, where the robot acts in opposition to the wearer’s movements (Marchal-Crespo and Reinkensmeyer, 2009). Please refer to Section “Control” for further details. In all three modes, the torques applied to the user are relatively low compared to lower limb or power augmentation scenarios. This is also true in the case of upper limb exoskeletons for teleoperative haptic applications, which need to generate feedback torques toward the user (Mallwitz et al., 2015; Buongiorno et al., 2019). It is worth mentioning that, although the assistive torques that need to be generated are much smaller compared to those in lower limb or power augmentation applications, the structure of these exoskeletons may need to support significant load due to the “cantilever” effect generated when the arm is extended—for example, during reaching. Therefore, despite the low required net torque, the structures of these exoskeletons may become bulky due to the need to withstand considerable gravitational load. In addition to this, upper limb exoskeleton kinematics are generally far from trivial due to the high number of degrees of freedom of the human and the complexity of the shoulder joint (Figure 2e,f; www.reneu.robotics.utexas.edu/harmony-exoskeleton; www.sms.hest.ethz.ch/research/current-research-projects/armin-robot/ANYexo.html; Kim and Deshpande, 2017; Zimmermann et al., 2019). Therefore, this exoskeleton type may need dedicated designs to allow correct replication of biological kinematics and consequently facilitate their control (Perry et al., 2009). In contrast to the fully actuated and typically heavy upper limb machines addressed above, there is a growing interest in exoskeletons for arm support or gravity compensation in industrial applications (Spada et al., 2017; Kim et al., 2018a,b; Van Engelhoven et al., 2018; Pacifico et al., 2020; Goršič et al. 2020). These devices are typically passive and use elastic elements to provide the needed compensation torque.

Regardless of the application domain or body area, stiff exoskeleton designs may present wearability issues since mechanical stress is generated on the user’s skin due to the generation of shear forces at the interface point. These shear forces may occur when there is misalignment between the exoskeleton rotation axes and the corresponding human joints. This represents a nonnegligible aspect of stiff exoskeletons, where the joints’ centers of instantaneous rotation are often fixed while the human joints’ (e.g., knee or shoulder) centers of instantaneous rotation change their relative position during the motion (Veeger, 2000; Hollman et al., 2002). As a consequence, aligning a stiff exoskeleton to a human limb might lead to undesired stresses. The amount of these stresses depends on the quality of the alignment and the capability to maintain it during motion. To deal with this, researchers are increasingly adopting original solutions that allow the exoskeleton structure to self-align to the anatomical kinematics of the wearer using additional passive degrees of freedom (Ball et al., 2007; Toxiri et al., 2015; Näf et al., 2018), compliant actuation systems (Vitiello et al., 2013), or specific mechanisms (Stienen et al., 2009; Ergin and Patoglu, 2012). However, these solutions require additional space and volume and are thus not always feasible in portable devices.

### Soft Exoskeleton Designs

**Figure 3.**
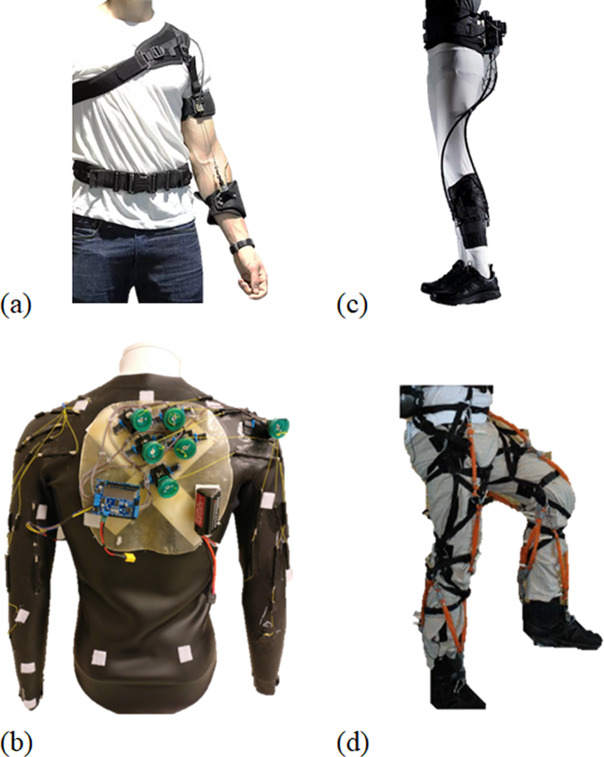
Examples of soft exoskeletons: (a) the research upper limb soft exosuit from Aries Lab (Xiloyannis et al., 2019), (b) the CRUX soft upper limb exosuit (Lessard et al., 2018), (c) the research lower limb soft exosuit from the Wyss Institute (Kim et al., 2019a,b), and (d) a lower limb pneumatic soft exoskeleton (Wehner et al., 2013).

In contrast to stiff devices, soft exoskeletons (also called exosuits) deliver mechanical power to the user using anchors that are typically made of clothing textiles and powered by cable-driven actuation (Awad et al., 2017; Schmidt et al., 2017; Li et al., 2018; Di Natali et al., 2019; Kim et al., 2019a,b; Xiloyannis et al., 2019; Yang et al., 2019; Goršič et al., 2021; Figure 3). Furthermore, soft exoskeletons are typically ergonomic, lightweight, and often designed following bioinspired techniques to better reproduce human physiological movements (Lessard et al., 2018; Figure 3b). They can be employed in several domains, from medical/rehabilitation scenarios to improving the physical strength of healthy individuals (Kim et al., 2019a,b; Figure 3c). The same work also demonstrated how a lower limb soft exosuit can reduce the metabolic cost of walking and running. Another important advantage is that, compared to stiff designs, exosuits largely maintain the natural freedom of movement of the wearer (Wehner et al., 2013; Figure 3d). However, the preservation of the freedom of movement offered by soft exosuits, as well as their comfort, depends on ergonomically appropriate design. Indeed, several recent works have focused on the design of ergonomic soft exoskeletons to minimize user discomfort (Andrikopoulos et al., 2015; Varghese et al., 2018; Choi et al., 2019; Chiaradia et al., 2021). These studies showed how the combination of textile fibers, 3D printed plastic materials and sometimes even metal parts, should be carefully designed to offer appropriate ergonomics and avoid possible limitations of the device’s functionality.

The main disadvantage of soft exoskeletons is that the mechanical power they can deliver is still limited as compared to stiff designs (Schmidt et al., 2017). This is mostly because the forces produced by soft exosuits inherently increase the shear stress on the human body. Indeed, although these devices may potentially offer higher comfort than stiff systems due to their compliant nature, soft exoskeletons often present problems of slipping and chafing at the anchor points due to the generation of shear forces at the interface point with the wearer (Asbeck et al., 2013; Yandell et al., 2017). On the other hand, the effects of the mentioned shear forces on joint loading are usually negligible: the radius over which they apply forces is significantly greater than that exploited by the biological tendon, and the joint compression force decreases as a consequence of this (Lamers et al., 2018). The shear force problem is instead minimized in stiff designs, where forces are applied orthogonally to the human segment (Yandell et al., 2017). Another common issue of soft design approaches is the friction introduced by the employed cable-driven actuation systems (e.g., Bowden-cable transmission), which typically results in low efficiency and bad control performance of the overall system if not compensated (Jeong et al., 2020). Furthermore, given their soft nature, the interface points where the human limb is anchored introduce a low-stiffness series compliance that would need high actuation power and appropriate bandwidth to be overcome (Chiaradia et al., 2019). Figure 3 shows examples of upper and lower limb soft exoskeletons.

### How Can Design Choices Facilitate the Adoption of Exoskeletons?

The mentioned size/weight issues typical of stiff designs have motivated a growing number of research groups to adopt methods from the field of industrial design to truly understand user needs and design exoskeletal solutions tailored to the specific application (Schmidt et al., 2017; Laffranchi et al., 2018). In most of these works, the main user needs that still need to be overcome are diverse: reduced donning time, lighter structures, higher autonomy of use, higher freedom of movement, increased comfort, and so forth (Gorgey, 2018; Wang et al., 2018; Fritz et al., 2019; Davis et al., 2020). Researchers and companies are addressing the needs with different solutions. One approach is the utilization of high-performance materials such as carbon fiber, aluminum or aluminum–titanium alloys, which may enable the development of lighter and more flexible structures for both upper and lower limb exoskeletons, thus reducing the overall weight of the device (Frisoli et al., 2005; Meijneke et al., 2014; Bianchi et al., 2016). A few devices propose modular architectures that allow the exoskeleton structure to be disassembled into multiple small and light modules to facilitate donning, transportation and autonomous usage by impaired subjects (Laffranchi et al., 2018; Vassallo et al., 2020; http://www.indego.com/indego/us/en/home). Finally, recent works show a growing interest in passive exoskeletons (Spada et al., 2017; Kim et al., 2018a,b; Van Engelhoven et al., 2018; Pacifico et al., 2020). As these devices are typically lighter than their active counterparts, they may be able to solve the weight issue. However, as their purpose is to counteract gravity in semi-static scenarios by means of passive mechanical components, they lack the versatility of active devices.

Regarding soft exoskeletons, their application is still limited to cases where the amount of needed assistance is limited—as in “low-torque” stiff exoskeletons—due to the problems mentioned in the previous section. A limited torque generation does not necessarily represent a constraint in the application of such devices, as seen in gait assistance exoskeletons where full torque compensation is not required. Nonetheless, it can be argued that considerable efforts are still needed to design appropriate ergonomics and realize systems that can effectively increase their load capacity without generating high stress on the wearer’s biological tissues (Lotti et al., 2020).

The search for novel solutions, both in stiff and soft exoskeletons, may be facilitated by careful analysis of users’ feedback and the inclusion of a diverse range of design disciplines besides engineering. Indeed, many innovative works are driven by approaches that dedicate considerable effort to understanding user needs and proposing solutions that are tailored to specific end-user needs. For example, user-centered design techniques have been applied to increase adoption of exoskeleton technologies in applications such as medical (Almenara et al., 2017; Laffranchi et al., 2018; Meyer et al., 2019; Vassallo et al., 2020), industrial (Babič et al., 2017; Davis et al., 2020), and assistive (Wang et al., 2018). Such user-centered design approaches, which may additionally incorporate industrial and ergonomics design in the development process, could serve as useful and effective tools to foster the adoption of exoskeleton technology (Hill et al., 2017).

A final consideration must be given to hybrid exoskeletons. The aim of these devices is to combine the light weight and comfort of soft exoskeletons with the generally higher torques generated by stiff solutions. Hybrid exoskeletons have been developed for both the upper and lower limb. For the upper limb, Rose and O’Malley (2019) proposed a hybrid hand exoskeleton that favors ergonomics, self-donning and doffing while satisfying the required range of motion and grasping force. Instead, for the lower limb, the reader might refer to the MyoSuit device, which demonstrated effective hip assistance without altering gait kinematics (Schmidt et al., 2017; Haufe et al., 2019). Moreover, an interesting hybrid knee exoskeleton with considerable high-torque capabilities has been proposed by Su et al., and showed contained mass properties and high backdrivability (Yu et al., 2019). As of today, there are no general and quantitative guidelines on how to combine stiff and soft elements in hybrid exoskeleton designs. In fact, each stiff or soft subcomponent embedded in a given hybrid design inherits the same pros and cons of stiff or soft designs seen in previous subsections. Therefore, the successful combination (and selection) of soft and stiff components for hybrid exoskeletons depends on the requirements of the specific application.

## Actuation

Actuators are a major bottleneck for the development of active wearable robots. The demanding requirements imposed upon them include high torque density (torque per unit mass), backdrivability, compactness and, for untethered devices, high energy efficiency. The actuator’s torque density is especially important for actuators at distal joints due to the large metabolic cost associated with carrying mass in these locations (Royer and Martin, 2005; Browning et al., 2007). Highly backdrivable actuators allow the wearer to move unimpeded when the robot is not providing assistance, for example, during the swing phase of walking or during donning and doffing. Finally, wearable robots should be as unobtrusive as possible. Actuators, which tend to protrude from the device, therefore need to be designed in small and compact form factors.

The majority of actuators for wearable robotics incorporate electric motors as prime movers (Windrich et al. 2016; Gopura et al., 2016; Veale and Xie, 2016). The main competing technologies, hydraulic and pneumatic actuators, are typically less efficient and reliable, tend to be noisy, and require pressurized fluid supplies that are difficult to make portable in an acceptable form factor (Madden, 2007; Veale and Xie, 2016). Hydraulic power units driven by combustion engines do have very high power densities, which makes them interesting when high power output is required, for example, for exoskeletons intended for load carrying and human performance augmentation (National Research Council, 2004). However, the high power density of hydraulic actuators comes at the cost of high impedance (Hollerbach et al., 1992; Zinn et al., 2004), which is undesirable for wearable robots. Overall, for most wearable robots that are not used for human performance augmentation, electric motors are considered to present the best trade-off between the different requirements.

Interestingly, in terms of power-to-mass ratio and energy efficiency, electric motors perform better than human muscles (Madden, 2007). However, in wearable robots, motors need to operate dynamically at varying torques and speeds. In these conditions, their efficiency is far below the nominal value (Verstraten et al., 2018). Moreover, the torque output of electric motors is low relative to the demands of wearable robots, which is why they are typically paired with high-reduction gearboxes. The dominant gearbox technologies in wearable robotics, harmonic gears and planetary gearboxes, offer high reduction ratios in compact sizes. However, their low efficiency and poor backdrivability are detrimental to the overall performance of the actuators and the wearable robot as a whole (Kazerooni, 1995; Nef and Lum, 2009; Lv et al., 2018; García et al., 2020).

Two paths are currently followed by the research community to tackle this problem. On one hand, novel gearbox technologies with improved characteristics are being explored, as summarized by García et al. (2020). Recent examples include the Gear Bearing Drive (Brassitos and Jalili, 2019), and the Bilateral Drive Gear (Matsuki et al. 2019). These gearboxes achieve high gear reductions at high efficiencies and in compact sizes through the use of the Wolfrom gearbox topology, combined with performance-enhancing modifications such as a carrierless design (Gear Bearing Drive) and profile shift modification (Bilateral Drive). On the other hand, quasi-direct drive has emerged as a new actuation paradigm. It combines high torque-motors with low-reduction gearboxes, resulting in energy-efficient actuators with high backdrivability (Sensinger et al., 2011). Ideally, these gearboxes are single-stage (gear ratio below 10) since efficiency is mostly related to the number of stages. Following their success in legged robots (Seok et al., 2015; Ding and Park, 2017), quasi-direct drive actuators are currently being implemented in wearable robots (Wang et al., 2018; Elery et al., 2020; Yu et al., 2020; Zhu et al., 2021).

To further compensate for the inherent limitations of commercial motors and gearboxes, researchers have resorted to specialized actuator designs. Our musculoskeletal system is built in such a way that elastic tissues in our body (tendons, aponeuroses, elastic tissues between muscle fibers, etc.) can perform a large amount of work by passive stretch and recoil (Alexander, 1990). This is possible because the joint torques applied during human movements typically exhibit a linear relationship with respect to the joint angle during consistent parts of the movement (Feldman, 1980; Hasan, 1986; Frigo et al., 1996; Toxiri et al., 2015). Engineered actuators for wearable robots, which are designed to provide assistive torques with a similar profile, can therefore also benefit from incorporating elastic elements with well-chosen stiffness. These specialized actuators are called elastic actuators.

Springs with well-designed stiffness allow actuators to deliver peak powers well beyond the capabilities of the motor itself (Paluska and Herr, 2006). Elastic actuators can therefore incorporate a smaller electric motor and gearbox, resulting in an overall reduction of weight and size compared to a traditional, stiff actuator. Moreover, springs offer the possibility to mechanically store energy when the actuator performs negative work and release it when positive work is required. As such, the average power flow through the actuator is reduced, resulting in an improvement in energy efficiency of the actuator (Hollander et al., 2006; Verstraten et al., 2017; Bolivar Nieto et al., 2019).

Elastic actuators can be subdivided into two main categories based on the placement of the spring element. By adding a (unidirectional) parallel spring to a stiff motor, one obtains a parallel elastic actuator (PEA). In this case, the parallel spring takes over a part of the load, leading to a reduction of the motor torque. The required motor speed remains unchanged since the motor is directly coupled to the output. Another option is to place the spring in between the motor and load, that is, in a series configuration. This type of actuator is called a series elastic actuator (SEA). In contrast to the PEA, a well-tuned spring lowers the speed required from the motor while the motor torque is unaffected. Although SEA and PEA provide similar levels of peak power amplification and increased energy efficiency during locomotion tasks (Verstraten et al., 2017), the PEA has the specific ability to compensate for static torques by tuning the equilibrium angle of the spring (Beckerle et al., 2016). This makes the PEA particularly suitable for upper-body exoskeletons that assist with overhead work or back-support exoskeletons for lifting tasks. Here, the parallel spring can provide the torque required to support the weight of the arms or torso as well as the load.

The performance of elastic actuators can be further enhanced by adding locking mechanisms and clutches (Plooij et al., 2017). Passive occupational exoskeletons often allow the user to disengage the spring providing the assistance so that it does not impede the user by generating a torque when it is not needed (Zhang and Huang, 2018). In a similar fashion, since little torque is required during the swing phase of gait, lower limb exoskeletons and prostheses can benefit from clutched PEAs (Haeufle et al., 2012; Geeroms et al., 2017) and SEAs (Rouse et al., 2014; Convens et al., 2019) that only engage the spring during stance. In robotic ankles, a catapult-like mechanism consisting of a spring and locking mechanism could be used to deliver the explosive burst of power required during push-off in an energy-efficient way (Collins and Kuo, 2010; Cherelle et al., 2014). Clutches have also been implemented in new actuator designs with multiple springs, which can be recruited progressively to contribute to the output torque (Mathijssen et al., 2015; Krimsky and Collins, 2020).

Most springs are manufactured to exhibit a linear relationship between torque and deflection. However, in wearable robotics, the required torques are rarely perfectly linear with respect to the output angle over the entire operating range of motion. The joint stiffness profiles required for walking, for example, exhibit low stiffness at low joint flexion and high stiffness at high joint flexion (Seyfarth et al., 2006). A closer match between the spring characteristic and the required torque can be achieved by means of nonlinear transmissions. The simplest way to generate a nonlinear transmission is by providing torque to the joint through a lever (Sugar and Holgate, 2013). The linear actuators driving the lever often consist of brushless DC motors combined with ball screw drives, a solution which offers relatively high reductions at high efficiencies. Other common nonlinear transmissions are cams (Realmuto et al., 2015; Gao et al., 2018) and four-bar mechanisms (Bergelin and Voglewede, 2012; Hyun et al., 2017).

Specialized actuator designs achieve impressive performance by relying on springs and nonlinear transmissions optimized for a specific task. The downside of this approach is significantly worse performance in off-design conditions. This issue can be mitigated by variable stiffness springs (Vanderborght et al., 2013; Shepherd and Rouse, 2017), variable transmissions (Mooney and Herr, 2013; Tran et al., 2019), multispeed gearboxes (Sugar and Holgate, 2013) or multimotor solutions (Verstraten et al., 2019), which allow the relationship between the required output torque and the spring characteristic to be shaped depending on the task. However, these solutions require additional motors or mechanisms, which increase the weight of the actuator. The metabolic penalty resulting from the additional weight can be avoided by remote actuation through, for example, cable- or wire-based transmissions, as implemented in several rehabilitation exoskeletons (Veneman et al., 2006; Mao and Agrawal, 2012). In portable devices, these transmissions can decrease metabolic cost by enabling a more proximal placement of the actuators, near the human’s center of mass (Asbeck et al., 2015).

Motor and gearbox technologies continue to improve and these improvements will promote further adoption of wearable robots in the near future. However, commercial devices for specific and simple tasks (e.g., occupational exoskeletons) may very well continue to rely on passive solutions until actuators with superior torque densities and energy efficiencies become available. The major breakthrough may arrive through the development of radically new actuator technologies such as dielectric elastomer actuators (Veale and Xie, 2016). Until these technologies reach a mature state, we expect that advances will most likely come from specialized actuator designs as those described in this section.

## Sensing

Wearable robots require advanced sensors so that they can determine the user’s actual and intended motions, determine the state of the robot itself, and use this information as a basis for robot control. Any limitations in the sensing lead to suboptimal control, which limits the overall performance of the wearable robot. These limitations can occur at one of two levels: at the lower level of individual sensors and at the higher level of multisensor fusion.

### Sensor Hardware and Low-Level Processing

While wearable robot sensors suffered from significant noise-related issues in the past, extensive work has been done to address them. Challenges on the level of individual sensors now primarily involve addressing remaining gaps in knowledge related to specific sensor modalities, particularly those that are just becoming widespread in the field.

Wearable robot systems commonly include standard robot sensors integrated into the robot, such as joint encoders and strain gauges (Huang et al., 2019; Singer et al., 2020). Assuming that the robot is tightly attached to the wearer and its morphology matches the wearer, we can assume that human body kinematics can be obtained from robot body kinematics via a constant transformation (Sposito et al., 2020). Commonly, pressure sensors are also built into points where the robot touches the wearer, allowing interaction forces between the wearer and robot to be measured. While these sensors have traditionally been rigid, soft sensors are becoming increasingly common in wearable robotics, potentially providing better robustness and comfort. For example, Chiaradia et al. (2018) used soft strain sensors to monitor robot joint angles instead of traditional rigid encoders, and Kim et al. (2019a,b) combined deep learning with a full suit of soft strain sensors to extract full-body kinematics. Most soft strain sensors are resistive (i.e., change resistance in response to strain), though capacitive and optical sensors have also been developed for wearable robotics (Atalay et al., 2017; Jin et al., 2020; Kim et al., 2020). Such soft sensors will probably never entirely replace rigid sensors (since soft technologies are not needed in all applications), but are likely to be a major aspect of the next generation of wearable robotics. One limitation they currently face is that the soft sensors generally still need to be connected to “hard” electronics, though such electronic components will likely become more integrated into soft materials over the next few years as well (Sanchez et al., 2021).The weakness of sensors built into the robot is that they only obtain knowledge about wearer body parts that are attached to the wearable robot and that they require the wearer to either already move the robot (in the case of position sensors) or apply forces to the robot (in the case of force sensors) before the robot can react, reducing its assistive benefits. The latter weakness can be reduced by reducing the amount of wearer force/movement required to trigger robotic assistance, but this may cause false positives (Novak and Riener, 2015; Tucker et al., 2015). To obtain a more complete picture of the wearer, wearable robots are thus commonly augmented with two types of sensors: inertial measurement units (IMUs) and electromyography (EMG) sensors.

IMUs consist of accelerometers, gyroscopes, and (optionally) magnetometers, whose outputs can be combined to obtain IMU orientation. IMU technology is cheap and can be easily attached to wearers; thus, it is commonly placed on parts of the body not connected to the wearable robot—for example, in a leg exoskeleton, an IMU can be placed on the arm or trunk (Lazzaroni et al., 2020). Alternatively, the IMUs can be embedded in the robot itself, providing an alternative to classic robot sensors (Naseri et al., 2020). As the individual components of the IMU tend to have noisy outputs, obtaining IMU orientation requires the use of techniques such as Kalman filtering (Nazarahari and Rouhani, 2021). IMUs that incorporate magnetometers are generally accurate in magnetically homogenous environments, as the magnetometer provides a reference estimate of magnetic north. In real-world environments with magnetic disturbances, however, IMU noise increases significantly and requires the use of advanced compensation algorithms (Slajpah et al., 2017; Wittmann et al., 2019). These algorithms are present in high-grade IMU systems and can be transferred to lower-cost IMUs, but are not present in such off-the-shelf devices by default.

EMG sensors measure the electrical activity of human muscles using electrodes placed on the skin. EMG levels are proportional to muscle contraction levels, but appear prior to mechanical contraction, allowing the wearer’s intentions to be anticipated (Novak and Riener, 2015). EMG can also be combined with measurements such as joint angles to obtain estimates of muscle dynamics and torque (Lotti et al., 2020). Furthermore, if the wearer is too weak to move their limb themselves, EMG may still allow motion intention to be detected and the wearable robot to be moved accordingly (Yun et al., 2020). Additionally, EMG can be used as an effectiveness measure of the wearable robot: since wearable robots are meant to support the wearer and reduce human workload, reductions in EMG level can be considered proportional to reductions in human muscle demand (Goršič et al., 2021; Kermavnar et al., 2021). However, EMG measurements also suffer from significant variability, both intersubject (due to, e.g., different fitness levels) and intrasubject (due to, e.g., fatigue and sweat); they are also prone to noise due to mechanical movement and electromagnetic interference. Methods to reduce noise have been demonstrated in the literature (Gokcesu et al., 2019), but not adequately transferred to wearable robotics. Similarly, alternatives to EMG such as mechanomyography and force myography (Xiao and Menon, 2019) have promise as a less noisy measurement approach, but have seen limited use in wearable robotics.

Aside from these common sensor modalities, many others have been used in wearable robotics to a lesser degree but suffer from low accuracy, difficulty of incorporating into real-time control, or simply lack of evidence. For example, brain measurements (e.g., electroencephalography [EEG]) can be used to infer the wearer’s intended motions without any muscle activation, which could be used to control wearable robots for people with disabilities who cannot move on their own. Unfortunately, such brain measurements provide only limited information (e.g., left vs. right arm movement), and their utility as a stand-alone method is thus questionable except for severely paralyzed individuals (AL-Quraishi et al., 2018); however, they could potentially be combined with more popular modalities such as EMG (Li et al., 2019). As another example, heart rate, respiration and other physiological measures are commonly used as a metric of wearer workload (physical or mental) and thus robot performance (Torricelli et al., 2020), and could be used to adapt the amount of robot assistance; however, while studies have suggested complex approaches to doing this (Schürmann et al., 2019), only limited work in this area has been done, mostly with stationary wearable robots (e.g., the Lokomat) (Koenig et al., 2011a,b).

Finally, a particularly promising case of underexplored sensors should be acknowledged: all the above sensors are meant to obtain information about the wearer. However, sensors for wearable robots should also obtain information about the environment—for example, possible obstacles in the path of a person wearing a leg exoskeleton, or objects that a person wearing an arm exoskeleton may wish to manipulate. Work on such “exteroceptive” sensors has been done in multiple areas of wearable robotics. For example, in lower limb prosthetics, researchers have used depth sensors to identify obstacles in the user’s path (Liu et al., 2016; Zhang et al., 2019) and segment stairs in the user’s path (Krausz et al., 2015); similar approaches have been used in lower limb exoskeletons (Laschowski et al., 2020) and upper limb prosthetics (Shi et al., 2020; Mouchoux et al., 2021). Nonetheless, further work is critically needed to incorporate environment sensing into wearable robot sensing and control.

### Sensor Fusion

Sensor fusion refers to the process of combining data from multiple sensors into an estimate of the user’s motor intention or physical state; the main challenge with such fusion is that existing methods are not sufficiently robust for real-world use. Existing algorithms range from simple thresholding (e.g., activate assistance if user bends forward) to more complex approaches based on classification and regression using machine learning (Novak and Riener, 2015; Tucker et al., 2015). For example, classifiers can learn to identify desired gestures from EMG-based on a training dataset of previously recorded and manually labeled EMG data (Yun et al., 2020); similarly, regression algorithms can learn to estimate desired robot torque from ground reaction force (Naseri et al., 2020) or EMG (Gui et al., 2019).

Such classification and regression have achieved high accuracies in laboratory experiments; however, the high accuracies in laboratory experiments (where conditions are largely stable and only a small set of wearer behaviors is expected) tend to not generalize to more chaotic real-world environments. For example, a wearable robot designed to support lifting tasks can, in a laboratory environment, simply classify wearer behavior as “lifting” or “not lifting,” and provide assistance only when the “lifting” class is detected (Hlucny and Novak, 2020). In the real world, however, nonlifting behaviors can include a variety of actions, many of which may appear similar to lifting (e.g., squatting or stretching the arms). One possible solution is to first classify a common denominator movement, such as a pre-lift and in later stages distinguish lifting versus squatting (Jamšek et al., 2020). Alternatively, a multilevel sensor fusion algorithm is needed to first categorize the wearer’s behavior into broad classes, then more specific actions—for example, the three-level sensor fusion and control system proposed by Lazzaroni et al. (2020) for back exoskeletons or the multilevel stumble detection system proposed by Zhang et al. (2011) for artificial legs. In such multilevel sensor fusion systems, the different levels may be based on different sensor types—for example, using information about walking terrain to switch between different EMG-based classifiers in a prosthesis (Liu et al., 2016).

Additionally, advanced sensor fusion for wearable robots will likely need to incorporate aspects such as active and semi-supervised learning—the wearer may perform an action that the robot does not understand, and the robot must then decide whether to actively query the wearer about what they are trying to do (which may interrupt the intended action) or try to identify the action on its own (which may be inaccurate). In both cases, the robot then needs to adapt its sensor fusion rules so that it can deal with this new motion in the future. This may involve a significant but reasonable adaptation—for example, if the baseline of one of the sensors has drifted and the sensor data are thus a shifted version of previously seen data. In such cases, several authors have presented online learning strategies that can adapt robot sensor fusion rules (Spanias et al., 2018; Gui et al., 2019; Hu et al., 2019; Huang et al., 2019; Zheng et al., 2019). It may, however, involve a major change to the sensor fusion rules—for example, the observed action may be an entirely new type of motion, and the robot may then need to add it to its “dictionary” of possible motions. This challenge of dealing with numerous possible wearer behaviors, some of which may be unknown to the robot, represents a major challenge in wearable robotics, and improvements in this area have an immense potential.

Finally, advanced sensor fusion for wearable robots will need to consider advanced planning that does not rely on physical contact between the robot and environment. Such advanced planning can be done using, for example, the depth sensors discussed in the previous section, but remains largely underexplored (Krausz et al., 2019).

Wearable robots have made enormous strides in sensing hardware and software. Sensors can be unobtrusively integrated into the robot and/or placed on the wearer, and information from diverse sensors can be integrated into a reasonably accurate estimate of the wearer’s current and intended activity as well as physical state. While improvements can be made on the level of individual sensors (e.g., reducing IMU noise in magnetically nonhomogenous environments), we believe that the greatest gains can be made through broader adoption of less popular sensor modalities (e.g., environment sensors) and on the level of sensor fusion (e.g., multilevel sensor fusion and semisupervised learning). Such improvements will allow wearable robots to be effectively used in dynamic, unpredictable environments where they can have a major effect on human wellbeing and productivity.

## Body Interface

Wearable robots cannot provide any benefits if humans do not use them. State-of-the-art wearable robots can effectively reduce workload; however, limitations related to user comfort and acceptance are a major remaining barrier to widespread use (Kermavnar et al., 2021), together with high cost. It was recognized in early exoskeleton research that a certain amount of discomfort was experienced by the users (Abdoli-E and Stevenson, 2008; Wehner et al., 2009), while newer models have been reported to minimize this discomfort to a tolerable level (Sirawattanakul and Sanngoen, 2020; Kermavnar et al., 2021). Some wearable robots are heavy and bulky (Stadler et al., 2016; Näf et al., 2018; García et al., 2020), which can interfere with the user’s freedom of movement. Even with lighter models, a certain level of discomfort and movement hindrance has been reported (Baltrusch et al., 2018; Goršič et al 2020). Inevitably, the unloading of one part of the body results in an increased loading (i.e., force) on another. In spinal exoskeletons, the reduction of the load to the back is typically achieved via increased loading to the lower limbs, which can be sometimes reflected in increased leg muscle activity (Luger et al., 2021) and discomfort to the chest area (Hensel and Keil, 2019), although back exoskeletons may also reduce leg activity (Bosch et al., 2016; Jeong et al., 2020). In comparison, upper limb exoskeleton interfaces can increase the loading to the spine (Weston et al., 2018). This trade-off, as well as the effects of wearable robots on task performance, discomfort and perceived freedom of movement, must be taken into account when designing wearable robots and studying their effects. While these functional and subjective aspects of wearable robots are increasingly being addressed, research is predominantly done in laboratory environments with healthy young male participants (Kermavnar et al., 2021). Therefore, on-field research with different groups is needed to support the development of body interfaces, suitable for broader population. Interface design has also begun to address the issue of load transfer to other body parts. One possible way to address this is by extending the legs of the exoskeleton all the way to the ground. For instance, a recent prototype of a semi-active exoskeleton, designed in this fashion, has been reported to decrease the activity of both back and leg muscles (Wang et al., 2021). Novel exoskeletons such as the Guardian XO (CRI Writers, 2018), are also being developed to support immense loads without stressing any human body parts. On the other hand, back exoskeletons with good design seem to decrease the load to the legs in addition to unloading the back (Bosch et al., 2016; Jeong et al., 2020).

Exoskeleton interfaces should enable efficient transmission of mechanical power without compromising user comfort. Langlois et al. (2018) have proposed three crucial design requirements that need to be fulfilled to achieve this. First, the attachment points of the device on the human body segments should be as distant to the joint as possible to reduce interaction forces (e.g., attachment points of the hip exoskeleton on the thighs should be as close to the knees as possible). At the same time, contact areas should generally be maximized. However, caution is needed as some parts of the body may be more sensitive to pressure and increasing the surface area may not be desirable in such cases. Next, the exoskeleton should be designed in a way that prevents interface migration (e.g., sliding along the user’s body), as misalignments caused by sliding commonly reduce power transmission efficiency (Langlois et al., 2018). Power transmission is also increased with larger contact point stiffness (Asbeck et al., 2015; Langlois et al., 2020). This can be achieved by placing the contact points over the bony prominences, such as tibial surface in case of lower limb exoskeletons (Mooney and Herr, 2016) or pelvis bones in case of trunk exoskeletons (Näf et al., 2018). Alternatively, the pads can be tightly attached to the user, but this can cause discomfort. Keeping in mind the importance of contact point placement, prevention of interface migration and contact stiffness, the compactness of the design should not be overlooked. For instance, the additional inertia due to the device’s weight can increase actuation demands (Langlois et al., 2018).

Wearable robots for industrial environments are usually designed primarily with a focus on load reduction during strenuous physical tasks such as load lifting, overhead work and trunk bending (de Looze et al., 2016; Bao et al., 2019). However, to allow efficient work and freedom of movement, the interface should not interfere heavily with other movements such as walking, stair climbing and sitting down or standing up. Indeed, wearable robots often decrease performance in tasks that require quickness and range of motion (Kermavnar et al., 2020). While these aspects were partially addressed in earlier research (Wehner et al., 2009; Bosch et al., 2016), assessments involving subjective reports have only recently been introduced (Kozinc et al., 2020a). Such subjective reports allow potential drawbacks of specific wearable robots, especially those related to the interface and discomfort, to be identified. For instance, specific contact points that exert pressure on the user or limit their range of motion can be identified and considered in subsequent design (Abdoli-Eramaki et al., 2007; Baltrusch et al., 2018; Baltrusch et al., 2021; Kozinc et al., 2020b). Identifying the drawbacks of state-of-the-art devices should help developers optimize subsequent models. One recent example of approaches to improve the exoskeleton interface is found in the SPEXOR spinal exoskeleton. Specifically, a self-aligning mechanism was integrated into the hip joint, which prevents the sliding of the interface (Näf et al., 2018; Babič et al., 2019). With such improvements, exoskeleton interfaces could become more user-friendly. Another possible consideration for interface improvement is the option to adjust and switch off the exoskeleton’s support (Babič et al., 2019; Wang et al., 2021), which allows the user to freely and efficiently perform the tasks that do not require support (e.g., walking).

As mentioned, the unloading of one part of the body inevitably results in an increased loading somewhere else. For instance, the mechanism by which spinal exoskeletons contribute to reduced workload is generation of torque around the spine and/or the hips (Bosch et al., 2016; Stadler et al., 2016; de Looze et al., 2017, Näf et al., 2018; Babič et al., 2019; Kermavnar et al., 2020). Even though spinal exoskeletons are designed to compensate for relatively small amounts of required spinal torque (Näf et al., 2018), this unloading of the spine results in increased levels of force (and consequently pressure) on the exoskeleton attachment sites on the body (i.e., distal thighs and shoulder girdle). Similar observations have been reported for upper limb (Huysamen et al., 2018) and lower limb (Luger et al., 2019) exoskeletons. Therefore, it is crucial to consider discomfort and pain tolerance thresholds at typical attachment sites for wearable robotic devices, and to design the devices in a way that minimize the chances of these thresholds being reached. While many studies have investigated pain thresholds with algometry (Sterling, 2011), their relevance to wearable robotics is limited, as the surface of force application in case of standard algometry is typically set at 1 cm^2^ (Graven-Nielsen and Arendt-Nielsen, 2010). Potentially more useful evidence is found in studies using inflatable cuffs to induce pressure at different sites, typical for attachments of wearable robots to the human body (Kermavnar et al., 2018). There are only two studies to date that explore this issue directly. Yandell et al. (2020) applied the pressure to the human body with fabricated exosuit interfaces for the shoulders, thigh, and shank, and thereby provided normative data for discomfort thresholds. Similarly, Kozinc et al. (2021) applied a pressure using exoskeleton pads and straps to the thigh, pelvic and chest/shoulder areas. Exoskeleton developers are encouraged to consider these values during the design of wearable robots. One of the most important findings of both studies is the large (up to 10-fold) interindividual variability in discomfort thresholds. Thus, further studies should critically include sufficient sample sizes to encompass this variability. Additionally, an issue that remains largely unexplored is discomfort during longer-term use of exoskeletons (i.e., over a workday), where higher discomfort or pain levels might be seen due to temporal summation (Nie et al., 2005).

It is unquestionable that wearable robots have the potential to increase work performance and reduce physical loads to the body. Some exoskeletons with inferior design and poor kinematic structures and human interfaces have been shown to reduce freedom of movement and increase discomfort, which hinders widespread use. On the other hand, several models have achieved high levels of support without compromising overall user experience. Recent advances in methods to assess functional performance and discomfort, as well as the availability of normative data on discomfort tolerance, should help the developers to design better interfaces in the future.

## Control

When synthesizing controllers for advanced robotic systems, it is common to create a hierarchical controller scheme where high-, mid-, and low-level control actions are locally isolated from each other, thus providing a broader understanding of the control problem in question. Wearable robots and exoskeleton systems are no exceptions to that; it is important to separately plan these controllers on different levels so that interrelations between high-level decision-making, mid-level planning and low-level actuator control can be clearly identified (Jimenez-Fabian and Verlinden, 2012; Tucker et al., 2015). In this section, we succinctly cover low- and mid-level controllers, while high-level control is discussed in the next section.

### Low-Level Control

The central problem in low-level control is to achieve high-fidelity servo tracking for actuators. This problem can be addressed via two different strategies: (a) precise position control through the use of nonbackdrivable mechanisms and high-gain controllers, and (b) force and torque control. The latter has certain advantages in terms of safe and dependable physical human–robot interaction (Vallery et al., 2008) while the former can guarantee performance metrics if trajectory tracking is prioritized (Ohnishi et al., 1996).

On one hand, force-controlled systems may require hardware-level modifications in actuators, for example, deployment of custom-built springs or strain gauges (Kashiri et al., 2017) and thus lead to hardware complexity. On the other hand, position-controlled systems generally possess high mechanical impedance at the output and are therefore deemed inappropriate for wearable robots and exoskeleton systems other than except rehabilitation procedures such as passive trajectory tracking tasks (Brahmi et al., 2019). Although the list of pros and cons can be further extended, force controllability is essential to establish dependable human–robot interaction control (Haddadin et al., 2009).

To implement force control at the actuator level, most systems employ SEAs in which a spring is attached between the mechanical output and motor (Pratt and Williamson, 1995); see the Section “Actuation.” The direct use of spring deflection feedback leads to instability issues (Pratt et al., 2004). To obtain fine torque tracking with guaranteed stability, one could employ cascade control strategies (Vallery et al., 2007), sliding mode with disturbance observer (Sariyildiz et al., 2020), or other observer-based robust control methods that are readily available for online simulation (Ozcinar and Ugurlu, 2021; Ugurlu et al. 2021). To this end, observer-based techniques were proven to be effective in handling perturbations due to uncertainties and unknown environmental effects (Haninger et al., 2020).

The performance of low-level force control depends on several factors: for example, actuator bandwidth, whole-body system dynamics, sensor signal quality, inner loop (current, pressure, etc.) control bandwidth, unmodeled dynamics, and sampling rate. Yet, given a completed hardware setup, one of the major contributions of a control engineer is to obtain a sufficient description of actuator dynamics. Once a sufficiently descriptive mathematical model is obtained, one can achieve fine force tracking for robot joints with pneumatic muscles (Ugurlu et al., 2019), SEAs (Vallery et al., 2007, Yildirim et al., 2019), hydraulic systems (Otten et al., 2015) and variable impedance actuators (Kaneishi et al., 2018). Through a detailed mathematical model, sensorless torque control is possible even for robot joints with nonbackdrivable mechanisms (Ugurlu et al., 2015). With this in mind, we argue that the implementation of force control is possible for most of systems to an extent, although it may not be a trivial task.

In summary, low-level force control at the actuator level became a standard in the wearable robotics community and there is a significant amount of literature on the topic (van der Kooij et al., 2006; Veale and Xie, 2016). The use of SEAs is arguably the most common approach owing to its relatively simple mathematical model and practical feasibility. Regardless of the actuator type, it can be argued that there is no open research question in relation to low-level control; however, engineering problems do persist. For instance, the bandwidth of mid-level control strongly depends on the low-level controller (Buerger and Hogan, 2007); thus, physical low-level controllers with deterministic real-time capability and higher sampling frequencies (~5 kHz) are required. Moreover, the integration of force sensing elements always introduces certain technical challenges to be overcome (Ham et al., 2009; Kim et al., 2018a,b; Yildirim et al., 2021).

### Mid-Level Control

Mid-level controllers are mostly task-specific and built on the basis of realizable actuator commands. To this end, the output of a mid-level controller is usually a link torque or position command that must be attained by the low-level controller. As nearly all wearable robotics applications involve physical interaction, impedance/admittance control is widely used (Hogan, 1985). In basic terms, an impedance controller outputs a certain amount of force command to render a given mechanical impedance, thus requiring force control. An admittance controller performs the same duty; instead, its transfer function is the inverse of impedance controller and it outputs a certain amount of displacement that corresponds to the aforementioned force command to render mechanical impedance. In practical applications, admittance control is preferred mostly when force-controllable actuators are not available or the torque command is not accessible; however, its tuning is more challenging and usually entails complex algorithms (Zheng et al., 2019).

Regardless of the impedance/admittance formulation, both controllers enable us to manage the trade-off between position and force tracking through the rendered output impedance. Hence, wearable robots for walking assistance need to possess active impedance control to cope with ground reaction during touch-down (Veneman et al., 2007). When employed via impedance approach with highly backdrivable robots (Lv et al., 2018), or via admittance approach with nonbackdrivable robots (Ugurlu et al., 2020), the locomotion control performance sufficiently increases. However, in these locomotion scenarios, human subjects are considered to be passive and the robot completely handles the walking behavior, regardless of the user-intended forces.

When it comes to robot-aided rehabilitation in general, training paradigms are constructed either in assistive or resistive configurations (Marchal-Crespo and Reinkensmeyer, 2009). Assistive controllers usually exert additional torques in a way so as to help the user to complete the task. For instance, force field controllers can be used to attain assist-as-needed strategies (Banala et al., 2009). Intuitively speaking, an assistive controller is always necessary to help the user to carry out the desired tasks. On the contrary, some researchers argue that resistive controllers can provoke neuro-motor plasticity and are thus more favorable in robot-aided rehabilitation for patients that possess some degree of strength and control (Washabaugh et al., 2019). From a control engineering perspective, both strategies can be effectively achieved using various forms of impedance control and/or rendered force fields.

The mid-level control strategies vary significantly across different disciplines since impedance and force control techniques are generally tailored to meet task-specific requirements. These requirements vary from feasible and dynamically equilibrated locomotion controllers (Ugurlu et al., 2016; Ugurlu et al., 2020) to minimization of a user’s deficit in human–machine interaction (Fitzsimons et al., 2019). Therefore, synthesis of a mid-level controller requires the determination of task-specific needs in accordance with the target use of the wearable robot. The reader can refer to review articles for further information in this matter (Marchal-Crespo and Reinkensmeyer, 2009; Yan et al., 2015; Proietti et al., 2016; Tucker et al., 2016; Chaparro-Cárdenas et al., 2018).

## Human–Robot Interfacing and Coadaptation

Low-level control of wearable hardware is an essential basis for practically applicable exoskeletons. Moreover, exoskeletons can only successfully perform various tasks in synergy with their human users if they are able to adapt to the user while considering the fact that the user simultaneously adapts to the exoskeleton. To do this, a high-level control system is required to infer human intention and create and adapt reference actions to make sure that the desired task is executed as intended by the user and assisted as intended by the exoskeleton. On the other hand, for the human to understand the exoskeleton’s behavior, it is also important that it behaves consistently and predictably; thus, the high-level control system needs to be designed with human-in-the-loop aspects in mind.

If a wearable device, such as an exoskeleton, is to perform actions to accommodate and assist humans in performing their tasks, the high-level control system should be able to extract the human user’s intention. Several signals can be employed to infer the intention, and the high-level control system thus tightly connects to the sensing hardware and methods. While the Sensing section focused on how to obtain the signals, this section focuses on how to use the signals in the high-level controller in order to achieve effective interfacing and coadaptation between the user and the exoskeleton.

Since the human and wearable device are physically coupled at least to some degree, haptic sensing is a common choice to detect the intended actions of the user. Such signals can be obtained by force/torque sensors or pressure sensors that are mounted on or inside the exoskeleton and can measure the interaction forces between its structure and the user’s body (Kazerooni and Guo, 1993; Nagai et al., 1998; Kong and Jeon, 2006; Lee et al., 2014). When the exoskeleton contains elastic elements like springs, the interaction force and thus control intention can also be estimated indirectly through the position of the spring (Pratt et al., 2004). An alternative is to simply use the measured position or motion induced by the user (Kazerooni et al., 2006). Position and motion states can also be used to detect the human intention and then play out a specific predefined feedforward behavior of an exoskeleton (Kawamoto et al., 2003a; Varol et al., 2010; Li and Hsiao-Wecksler, 2013). Information about the Center or Pressure or Center of Gravity can be used to a similar end for walking tasks (Farris et al., 2014).

Another common type of signals used for high-level control involves measuring the human body’s electrical activity: for example, muscle activity by EMG (Rosen et al., 2001; Kawamoto et al., 2003b; Fleischer and Hommel, 2008; Huang et al., 2009; Kiguchi and Hayashi, 2012), brain activity by EEG (Soekadar et al., 2015; AL-Quraishi et al., 2018) and eye activity by electrooculography (EOG) (Soekadar et al., 2015). Such electrical signals are advantageous since they can provide the information slightly prior to the actual human movement and potentially even if the human cannot make a physical movement (Fleischer and Hommel, 2008). On the other hand, their main disadvantage compared to haptic or position signals is that they require probes to be attached to the user, which usually comes with complex equipment and time-consuming calibration procedures.

Based on the detected human intention, the exoskeleton can devise appropriate physical actions to adapt and assist the users in their tasks. The simplest method is to amplify a signal that represents the user’s effort. For example, the measured human interaction force in a given direction can be amplified by the exoskeleton to assist the motion in that direction (Kazerooni et al., 2006; Kong and Jeon, 2006). If the user induces motion in the desired direction, then the exoskeleton can derive the assistive movement through a dynamical model (Kazerooni et al., 2006). In case of EMG, the intention signal (i.e., muscle activation) first has to be transformed into force by biomechanical models of muscles before it can be amplified (Rosen et al., 2001; Fleischer and Hommel, 2008). However, biomechanical models are user-dependent and can be hard to obtain. In some cases, a proportional mapping between EMG-derived muscle activation and the resulting exoskeleton assistive force can be sufficient for practical applications (Kawamoto et al., 2003b; Ferris et al., 2006; Lenzi et al., 2012). In complex tasks like locomotion, effort amplification alone might not be sufficient and dynamical models might be required; furthermore, different phases of the task may require distinct control modes (e.g., separate mode for a stance phase and a swing phase) (Kazerooni et al., 2006).

If the high-level control system of an exoskeleton has an insight into more complex human biomechanical models, it can exploit them and consequently optimize specific features during assistance (i.e., besides generally reducing the user’s effort), thus making task execution more ergonomic. For example, limb and muscle manipulability properties change the ability of humans to produce endpoint forces and motions in different directions of Cartesian space. For example, a lower limb exoskeleton can use geometric limb manipulability to optimize for energy efficiency in walking (Kim et al., 2010). In another example, the muscle manipulability model can be used by an arm exoskeleton to keep the force and motion production capacity equal in all directions irrespectively of human arm configuration (Petrič et al., 2019).

More complex movements can be encoded by trajectories; however, they typically have to be preplanned or be of repetitive nature, like in rehabilitation scenarios (Tsagarakis and Caldwell, 2003; Riener et al., 2005). There are numerous ways to encode the trajectories, ranging from simple spline methods to more sophisticated methods involving nonlinear oscillators (Ronsse et al., 2011; Aguirre-Ollinger, 2013) or dynamical movement primitives (DMPs) (Huang et al., 2016; Peternel et al., 2016; Lauretti et al., 2018). These trajectories can be learned from EMG feedback (Peternel et al., 2016) or from recorded motion directly (Huang et al., 2016; Lauretti et al., 2018). However, unlike effort-amplification methods, methods that merely involve repetition of learned trajectories do not enable the desired online and real-time adaptation to the user.

It has been shown that the human neuromechanical system can adapt to exoskeletons (Galle et al., 2013; Gordon et al., 2013). Exoskeletons that to adapt to the human user can adaptively update their assistive behavior (e.g., joint torques or motion) to minimize the user’s effort (e.g., measured by muscle activity or interaction force) (Kawamoto et al., 2003b; Krebs et al., 2003; Riener et al., 2005; Balasubramanian et al., 2008; Huang et al., 2016; Peternel et al., 2016; Teramae et al., 2018). For example, in Peternel et al. (2016), torque trajectories are increased as long as the user has to produce some effort to perform the given task (e.g., movement); therefore, the learned trajectories in a steady state compensate for all underlying dynamics (i.e., human, robot, and task). If the dynamics change, the user has to compensate for the change to perform the given task, and the changed human effort signal again reshapes the trajectories in real-time, thus achieving coadaptation between the user and the exoskeleton (Peternel et al., 2016). Alternatively, reinforcement learning can be applied to optimize learned trajectories based on some specific metrics while the user performs the task (Huang et al., 2016). The main advantages of adaptive learning methods are that they can reshape complex trajectories in a real-time manner and, unlike direct effort amplification, do not require a constant user effort when the task movement or force is repetitive (Peternel et al., 2016).

As outlined above, several major fundamental principles for high-level control of wearable devices have been proposed in past decades. We predict that future research will mainly focus on improving upon existing fundamental principles, as has been already done in the recent years. Most of the fundamental human–robot coadaptation control methods still require various improvements in terms of processing speed and accuracy, control intelligence and adaptability, and design practicality in order to make them effective in realistic real-world scenarios. Nevertheless, some methods are also inherently reliant on research in complementary fields, especially sensing-related research to obtain meaningful and information-rich intention signals that can be used by the control system. For example, decoding brain activity (either through EEG or some other method) heavily relies on developments in the field of neuroscience, while EMG-based and model-based methods rely on developments in the fields of human motor control and biomechanical modeling. We believe that the most powerful and convenient way for the high-level control system to govern user-exoskeleton coadaptation would be to have a complete and detailed understanding of brain functions and a more practical means to measure it. However, as of now, we are still quite far from this point.

## Benchmarking

Demonstrating that a device is working properly for a given application and communicating this information to end-users and stakeholders is an essential task when developing a new product. Very often, the performance of an exoskeleton prototype is considered “demonstrated” after the execution of a few experiments in a laboratory using ad hoc sets of metrics, which are usually established by the same researchers involved in the development process. This common practice should be seriously reconsidered if the wearable robot community wants to promote rigorous comparison across existing systems as a basis of optimized and efficient product development (Torricelli et al., 2020), and benchmarking may help in this process.

Benchmarking is a comparative evaluation process often used by large companies to measure and improve the quality of products, services or processes. By means of comparisons with a standard, or benchmark, it is possible to identify the gaps in the state of the art and find strategies to improve competitiveness. But how can we translate this concept to wearable robotics? What features should be the object of comparison? Which devices or behaviors should be considered as standard or reference? The main problem is that exoskeleton research is still at an early stage, with hundreds of solutions at low technology readiness levels and only a few commercial solutions with unclear performance levels. One of the main mistakes in comparing robotic systems is to think in terms of “this robot is better (or worse) than that robot.” This viewpoint, common in competitions, does not reflect the multifactorial nature of performance. For instance, a faster system is not necessarily better than a slower one, and similar principles apply to many variables and features that may be used to describe the performance of a system. Nonetheless, comparing a characteristic of a system with the same characteristic of another system is still a very valuable process in order to understand why that system is behaving in a certain way. And this is what benchmarking is about: comparing to understand.

Recent efforts in the wearable robotics community made apparent the difficulty of defining the performance of a system on simple scales, triggering interesting discussions on the terminology to be used to define a given aspect of performance (Torricelli et al., 2020). What we understood from these discussions is that the evaluation of performance is strongly dependent on the robotic technology under examination. For instance, “interaction” may assume very different meanings if applied to an industrial manipulator rather than to a wearable robot. This also applies to other system abilities, for example, “adaptability” and “decisional autonomy.”. One of the first steps in benchmarking is to agree on the definition of these concepts with respect to the specific technology considered. The motor function and the application domain are two additional factors that influence the definition of performance. For instance, would “stability” mean the same for rehabilitation and industrial robots? Would a unique definition apply to walking and reaching skills equally? Without clear definitions, it becomes impossible or even worse, incorrect, to compare two systems.

Another crucial aspect of benchmarking is categorizing the multiple facets of a given system into meaningful subgroups. Some years ago, Torricelli et al. (2015) proposed to categorize the “motion abilities” of a bipedal system into four classes: efficiency, stability, kinematics, and dynamics. Each of them was composed of four to five quantitative indicators obtainable by direct measurements. Whether this classification is appropriate or not is yet to be proven. However, the methodological approach of decomposing the performance of a system into measurable items seems promising. Future research efforts aiming at identifying quantitative indicators for all the missing system abilities would be strongly valuable for the wearable robotics community. In this respect, one largely overlooked aspect is human–robot interaction, which is particularly difficult to characterize since it is strongly dependent on the subjective perception and experience of the user (Beckerle et al., 2017). Another crucial research direction in robotic benchmarking is establishing causal relations between the specific actuator typology and the overall performance in a given application. This would greatly help developers in identifying how improvements in components would contribute to better system behavior.

The discourse on system abilities and their definitions should be taken to a practical level to bring a real impact to the community. It is useless to design a meticulous protocol able to test a specific performance aspect very rigorously if such a protocol cannot be easily reproduced in different labs and systems. If benchmarking is about comparison, the reality of experimental settings should be considered. For this reason, the concept of “reproducibility” as opposed to “replicability” is worth clarifying. As well explained by Plesser (2018), reproducibility allows comparability of results from experiments conducted using different equipment (e.g., different sensors or environment) and even different procedural steps (e.g., a different number of trials). Replicability, instead, requires two tests to be executed exactly in the same way, using identical experimental setups, to produce comparable results. For benchmarking to be extensively adopted, we support the direction of reproducibility, even when this implies renouncing accuracy to a certain level. Very recently, several researchers have started to identify reproducible metrics, protocols and testbeds to quantify performance of exoskeletons in a wide range of motor skills and application domains (Torricelli and Pons, 2019; Baltrush et al. 2021; Kozinc et al., 2020a; Madinei et al., 2020; Pinto-Fernandez et al., 2020; Pesenti et al., 2021). This notable increase in scientific interest is being accompanied by the intense work of several international committees for standards (e.g., ASTM F48, ISO TC299, and CEN/CENELEC), which contribute to fill two important gaps: the availability of standardized testing methods and rigorous quantitative data on exoskeleton performance.

Nevertheless, even in the presence of agreed terminology, reproducible protocols and testbeds, and data-driven evidence, a missing element to ensure wide adoption of any potential standard testing methodology is the data format. Anyone with a clear and reproducible protocol and a proper experimental setup can run an experiment, obtain data and calculate the desired metric. But, in order to benchmark such system, a comparison with a reference should be done. If results cannot be easily shared and put together, for example, using unified databases, individual efforts in producing datasets are likely to remain limited to local (single lab-based) comparisons, or relegated to simplistic comparisons using easy-to-understand but poor metrics (e.g., speed and power consumption). To exploit all the potential that a unified database may provide, it is imperative to first find a common format for data, and second, make the existing computational algorithms to be compliant with such standardized data format. This way, anyone can compare their own system with another one, or with normative data obtained from all similar previously tested systems. To reach this situation, some bottlenecks should be overcome, the most important one being the regulatory constraints on sharing data given that tests with wearable robots intrinsically include a human in the loop.

## Conclusion

We have presented challenges related to seven important technological aspects of wearable robots that are currently limiting broader adoption of such robots in numerous everyday applications, from healthcare to sports. In the following paragraphs, we summarize the most important challenges that should be considered by developers of exoskeletons and other wearable devices.

Regarding mechanical design, many traditional exoskeleton structures and layouts tend to be obtrusive for the wearer during use and general management of the device. To increase the usability of these devices, researchers are working on novel solutions that target easier management through innovative design concepts: for example, modular structures, lighter machines specifically optimized to the degree of assistance that needs to be delivered, exosuits, and textile elements.

Actuation represents a major bottleneck for wearable robots, as their torque density, energy efficiency and backdrivability do not yet meet expectations. The inherent limitations of electric motors and gearboxes are the root of the problem. Improvements in these conventional technologies are slow, and promising alternative technologies are not yet mature. Specialized actuator designs incorporating springs, locking mechanisms, nonlinear transmissions and/or multiple motors can provide solutions, but introduce new trade-offs in terms of versatility, system complexity, and compactness.

In the area of sensing, the greatest challenge is in high-level sensor fusion. Such fusion has achieved good accuracies in controlled laboratory conditions, but these accuracies do not generalize to more uncontrolled real-world environments, which require additional work on multilevel and adaptive sensor fusion algorithms. Furthermore, additional improvements may be achieved through broader adoption of less common sensor modalities, such as exteroceptive environment sensing.

Limitations related to user comfort and acceptance are a critical remaining barrier to widespread use of wearable robots. Interfaces should not limit the comfort of the user and should simultaneously allow efficient power transition, which is achieved by optimal placement of the attachments to the human, larger contact stiffness, and prevention of interface migration.

Low-level and mid-level control architectures constitute a solid basis for high-level decision-making blocks, such as human–robot coadaptation. A possible performance degradation in low- and mid-level controllers may be directly reflected to overall system behavior, causing it to underperform the expected tasks. Therefore, one must construct a hierarchical control architecture first by ensuring the high-fidelity tracking performance at the inner control loops before gradually synthesizing the outer high-level controllers.

It is imperative that wearable devices perform tasks in synergy with their human users and simultaneously adapt to each other. Therefore, a high-level control system is a necessary element to infer human intention and create/adapt exoskeleton actions that facilitate the execution of the desired task as intended by the user. The high-level controller can infer the user’s intention based on various signals, each of them having pro and cons in terms of control.

Describing the performance of a wearable robot including its components is a multidimensional problem. Benchmarking is a methodology that can help researchers and developers to quantify the different facets of performance and allow truthful comparisons between systems. Several issues should be considered to convert benchmarking into a common practice in the wearable robotic community. The most relevant ones are: converging on agreed terminology, quantifying human–robot interaction, define reproducible protocols and testbeds, and identifying common and sharable data formats.

While we have focused on the main challenges and aspects that many wearable robot applications have in common, it remains important to be aware that eventual success of wearable robots will equally depend on their specific tailoring and adequacy to the specific application case, always demanding that all critical user needs associated with that specific use case have been met. Additionally, successful implementation of wearable robots in our everyday life also depends on equally or even more important ethical, legal, and societal questions that deserve attention of experts in the relevant fields.

## Data Availability

Data availability is not applicable to this article as no new data were created or analyzed in this study.

## Footnotes

1 Please note that ensuring mechanical contact of the machine structure with the ground is also needed in several types of upper limb exoskeletons, as will be explained later in this paragraph.
